# Changes in a Single Institution’s Orthopedic Hospitalization Service in Japan Owing to COVID-19 in 2020

**DOI:** 10.7759/cureus.14410

**Published:** 2021-04-10

**Authors:** Hirofumi Bekki, Takeshi Arizono, Ryuji Tagata, Akihiko Inokuchi, Takahiro Hamada, Ryuta Imamura

**Affiliations:** 1 Department of Orthopaedic Surgery, Kyushu Central Hospital of the Mutual Aid Association of Public School Teachers, Fukuoka, JPN

**Keywords:** coronavirus, covid-19 pandemic, hospital charge, economic impact, surgery

## Abstract

Introduction

The coronavirus disease 2019 (COVID-19) pandemic has had immense impact on people and institutions, including the number of admissions to hospitals for surgery. Our aim in this study was to determine the impact of the pandemic on surgeries in a single institution located in Fukuoka, Japan, between 2019 and 2020.

Methods

We quantified the numbers of surgeries in both years according to three sites of injury (indoor, outdoor, and unknown), 14 disease categories, and 9 primary diseases using patients’ medical records. We also compared the hospital cost per day in each month from March to November in both 2019 and 2020 and compared the change in these costs between the two years.

Results

The number of admissions in 2020 was 1,187 cases vs 1,282 cases in 2019. The average patients’ age was higher in 2020 vs 2019 (69.7 ± 0.5 vs 67.5 ± 0.5 years, respectively; p = 0.004), with no gender differences (2020: 705 women and 482 men; 2019: 716 women and 566 men). We found no significant differences in the number of admissions by month between 2019 and 2020. The percentages of outdoor injuries were significantly lower in 2020 vs 2019 (29.8% vs 37.9%, respectively; p = 0.004), and we found significantly different rates when comparing 2020 and 2019 for degenerative disease (42.6% vs 37.4%; p = 0.007), trauma related to falls (34.4% vs 30.2%; p = 0.02), chronic disease (1.9% vs 3.7%; p = 0.005), and sports injuries (0.8% vs 3.7%; p < 0.0001). The rate of sports-related injury was significantly lower in 2020 (1.6%) than in 2019 (7.7%) (p < 0.0001). The daily hospital charge was $10,517.09 (US dollars) in 2020 vs $11,225.32 in 2019, and the charges in the months of April and June were significantly higher in 2020 vs 2019 (p = 0.003 and p = 0.001, for April and June, respectively). Both the number and rate of upper limb fractures were higher in 2020.

Conclusions

The COVID-19 pandemic is affecting some hospitals’ revenue. Although the charges per day were sufficient in our institution in 2020, compared with 2019, some hospital beds were unused during this phase of the pandemic. Hospitals may increase the revenue by mixing both short-term and long-term patients’ hospital stays effectively.

## Introduction

The Japanese government declared a state of emergency in response to the novel coronavirus disease in April 2020, but the coronavirus disease 2019 (COVID-19) pandemic began affecting the public’s lifestyle earlier, after reports of a new virus arising in China [1]. The COVID-19 pandemic has produced serious concerns and challenges for the entire medical community because of the fear of disease clusters. An epidemiological study in Hong Kong reported a 44.2% decrease in the number of operations [2]. According to a survey from the Asia Pacific Spine Society, 58.5% of respondents reported reduced outpatient clinic hours and 74.6% reported reduced operation theater hours [3]. Besides orthopedic surgery, COVID-19 has had profound economic effects on radiology practices and dental practices [4,5]. These outcomes imply the necessity for appropriate management to adjust human and financial resources and to maintain clinical services. We hypothesized that comparing hospitalization service between 2019 and 2020 would be useful to address the related economic impact by efficiently managing hospital admissions and treatment during the COVID-19 pandemic. The purpose of this study was to quantify the disease’s impact on our hospitalization service and to suggest a possible solution for the economic impact caused by COVID-19.

## Materials and methods

**Figure 1:**
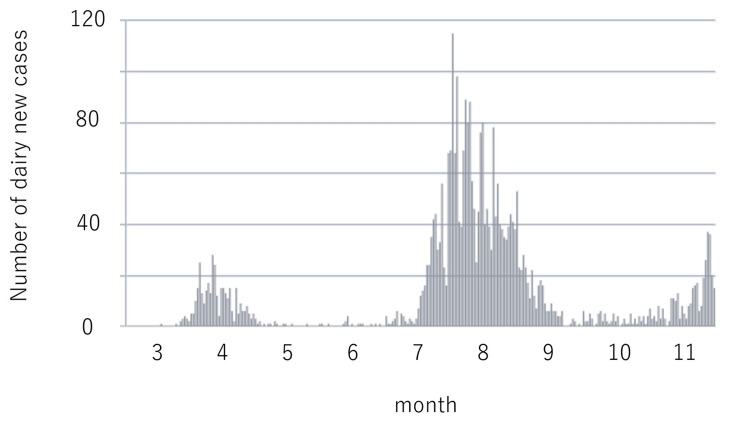
The number of daily new cases in our institution.

Clinical data from March to November 2020 were collected from the patients’ medical charts according to our institution’s ethics guidelines. This retrospective study was approved by the Institutional Review Board of the authors’ affiliated institution, Kyushu Central Hospital of the Mutual Aid Association of Public School Teachers, which is located in a city with a population of 1.6 million. The number of COVID-19 daily new cases is summarized in Figure 1 [6]. The city declared a state of emergency because of coronavirus disease for one month from April to May 2020. The government authorities made an appeal to the public for social distancing, and measures constituted school suspension, closure of amusement facilities, and the cancellation of social events. Because there were no infected staff in our institute, we did not limit the number of outpatients, examinations, and surgery, as in other hospitals [7].

Incidence sites

Trauma cases were categorized into three groups according to the incidence site: indoor injury, outdoor injury, and unknown injury site.

Primary disease

We classified all cases into nine categories: degenerative disease, trauma owing to falls, traffic accident-related injury, infection, chronic disease, iatrogenic disease, sports injury, unspecified or low-energy injury, and implant removal.

Treatment

We classified all treatments into 14 categories: non-operative, spinal decompression, spinal instrumental fixation, arthroplasty, osteotomy, arthroscopic surgery, fixation or reduction for upper limb facture or dislocation, fixation or reduction for lower limb fracture or dislocation, fixation for trunk fracture, amputation for infection, surgery for peripheral tendon or nerve injury, tumor resection, wound repair, and implant removal.

Hospital charges

Hospital charges, average revenue from a single patient, constituted medical fees for examinations, medications, intravenous transfusions, surgeries, and other expenses. This figure was divided by inpatient days, and the change in hospital charge per day was analyzed by month. We also evaluated the relationship between the charges and each treatment category. In this study, 100 Japanese Yen equaled 1 US dollar.

Statistical analysis

Data are presented as mean ± standard deviation. Differences between 2020 and 2019 were evaluated using Pearson’s chi-square test and Student’s t-test. The data analysis was conducted using the JMP statistical software package, version 9.0.2 (SAS Institute, Cary, NC, USA). A p-value of <0.05 was considered statistically significant. Confidence interval (CI) or odds ratio (OR) was also noted in association with p-value.

## Results

Patients’ demographic data

A total of 2,469 hospital admissions (1,187 cases in 2020 and 1,282 cases in 2019) were identified. Patients in 2020 were older than those in 2019 (average age: 69.7 ± 0.5 vs 67.5 ± 0.5 years, respectively; p = 0.004) (95% CI: 0.71-3.67), but the gender difference was similar (705 women and 482 men in 2020 and 716 women and 566 men in 2019). The third-quarter in 2020 showed an 8% decrease in the number of hospital admissions compared with 2019. The number of hospital admissions by month were as follows: In 2020, the number of admissions in March, April, May, June, July, August, September, October, and November was 163 (13.7%), 119 (10%), 118 (9.9%), 130 (10.9%), 132 (11.1%), 122 (10.3%), 135 (11.4%), 145 (12.2%), and 123 (10.4%), respectively. In 2019, the number of admissions for the same months was 158 (12.3%), 144 (11.2%), 138 (10.8%), 140 (10.9%), 151 (11.8%), 142 (11.1%), 130 (10.1%), 141 (11%), and 138 (10.8%), respectively. There was no significant change in the per month admission numbers between 2020 and 2019.

Incidence site

In 2020, there were a total of 547 cases (317 indoor injuries, 163 outdoor injuries, and 67 injuries from unknown sites). In 2019, there were a total of 595 cases (269 indoor injuries, 226 outdoor injuries, and 100 injuries from unknown sites). The rate of outdoor injuries was significantly lower in 2020 than in 2019 (29.8% vs 37.9%, respectively; p = 0.004) (OR=0.69).

Primary disease

A comparison of the primary diseases between the two years is summarized in Table 1. According to our classification, in 2020 vs 2019, there was no significant difference in traffic accident-related injury (2.9% vs 3.1%), infection (1.6% vs 2.7%), iatrogenic disease (0.4% vs 0.9%), implant removal (6.2% vs 6.6%), and unspecified or industrial accident other than falls (9.3% vs 11.6%). Comparing 2020 and 2019, respectively, we found significant differences in the rates of degenerative disease (42.6% vs 37.4%; p = 0.007) (OR=1.24), trauma owing to falls (34.4% vs 30.2%; p = 0.02) (OR=1.21), chronic disease (1.9% vs 3.7%; p = 0.005) (OR=0.49), and sports injuries (0.8% vs 3.7%; p < 0.0001) (OR=0.19). Limited to trauma, the rate of sports injury was 1.6% in 2020, which was significantly lower than the rate of 8.1% in 2019 (p < 0.0001) (OR=0.19).

**Table 1:** Comparison of primary diseases in 2019 and 2020.

Primary disease	2019, no. of cases (%)	2020, no. of cases (%)	p-Value
Degenerative	479 (37.4)	506 (42.6)	0.007
Trauma due to falling	387 (30.2)	409 (34.5)	0.02
Traffic accident	40 (3.1)	34 (2.9)	0.71
Infection	35 (2.7)	19 (1.6)	0.05
Chronic	48 (3.7)	22 (1.9)	0.005
Iatrogenic	11 (0.9)	5 (0.4)	0.18
Sports injury	48 (3.7)	9 (0.8)	
Unspecified or low-energy injury	149 (11.6)	110 (9.3)	0.06
Removal of implant	85 (6.6)	73 (6.1)	0.63

Treatment

The percentages of patients in the 14 treatment categories and in the non-operative category in 2020 and 2019 are shown in Table 2, and the comparison is as follows, respectively: non-operative, 34.9% vs 31.5%; spinal decompression, 15.7% vs 15.1%; spinal instrumental fixation, 5.1% vs 4.9%; arthroplasty, 6.6% vs 5.9%; osteotomy, 0.3% vs 0.6%; arthroscopic surgery, 1.6% vs 1.8%; fixation or reduction for upper limb facture or dislocation, 8.1% vs 6%; fixation or reduction for lower limb fracture or dislocation, 18.8% vs 18%; fixation for trunk fracture, 1.7% vs 1.8%; amputation for infection, 0.7% vs 0.5%; surgery for peripheral tendon or nerve injury, 1.7% vs 2.1%; tumor resection, 1.2% vs 1.1%; wound repair, 0.9% vs 0.8%; and implant removal, 6.2% vs 6.6%. The rate of fixation or reduction for upper limb facture or dislocation differed significantly between 2020 and 2019 (p = 0.04) (OR=1.38).

**Table 2:** The percentage of 14 treatment categories in 2019 and 2020.

Treatment	2019, no. of cases (%)	2020, no. of cases (%)	p-Value
Nonoperative	447 (34.9)	374 (31.5%)	0.08
Spine			
Decompression	193 (15.1)	186 (15.7%)	0.67
Instrumentation	63 (4.9)	60 (5.1%)	0.87
Joint			
Arthroplasty	75 (5.9)	78 (6.6%)	0.48
Osteotomy	8 (0.6)	4 (0.3%)	0.31
Arthroscopy	23 (1.8)	19 (1.6%)	0.71
Trauma surgery			
Upper limb	77 (6%)	96 (8.1%)	0.04
Lower limb	231 (18%)	224 (18.9%)	0.59
Trunk	23 (1.8%)	20 (1.7%)	0.84
Amputation for infected parts	6 (0.5%)	8 (0.7%)	0.49
Surgery for tendon nerve (hand, foot)	27 (2.1%)	20 (1.7%)	0.44
Tumor resection	14 (1.1%)	14 (1.2%)	0.83
Wound repair	10 (0.8%)	11 (0.9%)	0.69
Removal of implant	85 (6.6%)	73 (6.2%)	0.63
Total	1282	1187	

Hospital charges

**Figure 2:**
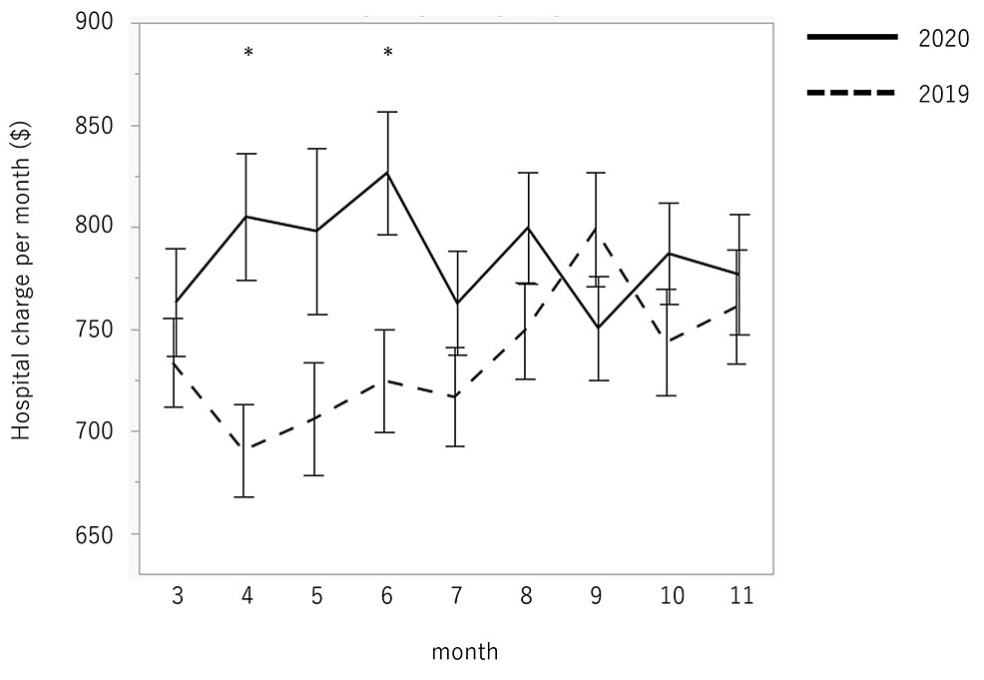
Changes in hospital charge per day. In April and June 2020, the charge per day was significantly higher than that for the same months in 2019. *p < 0.01.

**Figure 3:**
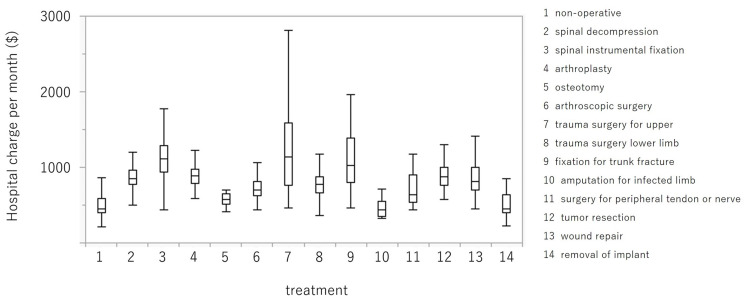
The average hospital charge per day by each surgical classification.

Total hospital charges were $10,517.09 (US dollars) in 2020 and $11,225.32 in 2019. The hospital charge per day in 2020 was significantly higher than in 2019 ($785.10 ± $9.60 vs $732.30 ± $8.50; p < 0.0001) (95% CI: $24.8-74.9). The changes in hospital charge per day are illustrated in Figure 2. The charges in March, April, May, June, July, August, September, October, and November 2020 were $763.00 ± $26.50, $805.00 ± $31.20, $798.00 ± $40.80, $826.40 ± $30.40, $762.60 ± $25.40, $799.60 ± $27.20, $757.30 ± $24.90, $787.00 ± $24.90, and $776.80 ± $29.60, respectively. The figures for the same months in 2019 were $733.50 ± $21.80, $690.60 ± $22.70, $705.90 ± $27.60, $724.70 ± $25.30, $716.70 ± $24.20, $749.4 ± $23.70, $798.70 ± $28.00, $743.40 ± $26.20, and $760.90 ± $28.00, respectively. In April and June, the charges in 2020 were significantly higher compared with the same months in 2019 (p = 0.003 and p = 0.001, respectively) (95% CI: $38.4-190.5 and $23.8-179.6, respectively). The average hospital charge per day by each surgical classification is shown in Figure 3. The charge per day for trauma surgeries for upper limb injuries was $1,206.80 ± $38.80, and this was the highest figure among the 14 categories, followed by $1,134.70 ± $25.00 for spinal instrumental fixation.

## Discussion

The purpose of this study was to quantify the impact on hospitalization services caused by COVID-19 in a single institution and to suggest a possible solution for the disease’s economic impact. Trauma surgeries for upper limb injuries were associated with good cost performance regarding hospital charges, but beds were not fully used owing to the decreased hospital admissions when comparing data for 2020 with data for 2019. Hospitals may increase their revenue by effectively mixing both short-term and long-term patients’ hospital stays.

The third-quarter in 2020 showed an 8% decrease in the number of hospital admissions compared with the same quarter in 2019. This figure was smaller than that reported in previous studies, and this may be because of differences in government policies and available inpatient beds [8]. According to our data for 2020, the relative rates of degenerative disease and trauma owing to falls were significantly higher in 2020 vs 2019, which differs from findings in a previous study [9]. Conversely, the rates of chronic disease, such as benign tumors or rheumatoid arthritis, and sports injuries were significantly lower. These results suggest that the public refrained from seeking treatment for injuries related to minimal trauma or chronic disease in 2020. Instead, the number of patients waiting for elective surgeries increased or patients asked for admission only when they experienced intense pain owing to falls.

Regarding the incidence site, the rate of outdoor injuries was significantly lower in 2020 than in 2019 (29.8% vs 37.9%, respectively; p = 0.004). Li et al. reported that falls occurred outdoors more often than indoors among most age groups [10]. Considering the older average age in our country and the higher rate of trauma owing to falls in this age group, people may spend more time indoors following “stay at home” initiatives, which meant that older people were more likely to fall indoors.

The absolute number and relative rate of treatment for upper limb fractures increased in 2020, whereas the values for other procedures were similar between 2020 and 2019. “Stay at home” initiatives may be associated with this outcome, and short-term hospital stays owing to upper limb trauma could have impacted the hospital charges, which led to the increased hospital charge per day in 2020. In addition, 18/77 (23.4%) surgeries for upper limb trauma in 2019 were performed in September and 6/96 (6.2%) in 2020 (data not shown). As shown in Figure 3, the change in hospital charges when comparing 2020 with 2019 was highest in September, which supports our interpretation.

A new variant of coronavirus was identified in the United Kingdom in 2020 [11], and the mutation made the SARS-CoV2 virus more infectious. New vaccine efficacy against COVID-19 is being urgently evaluated in clinical trials [12,13]. If a new pandemic arises in 2021, the different characteristics would invalidate current data. Nevertheless, to the best of our knowledge, this is the first study to clarify the impact of COVID-19 on surgical treatments from 2019 to 2020. Some medical institutes are facing difficulty surviving owing to the decreased revenue [14,15]. According to our output, hospital beds may be unused during this phase of the pandemic. To solve the risk of financial crisis, hospitals may increase the revenue by mixing both short-term and long-term patients’ hospital stays effectively. We hope that our findings help in the response to a possible next wave and help maintain hospital revenue by assisting with efficient admissions during the COVID-19 pandemic.

## Conclusions

Our aim in this study was to determine the impact of the pandemic on surgeries in our institution between 2019 and 2020. We found no significant differences in the number of admissions by month between 2019 and 2020. We found significantly different rates when comparing 2020 and 2019 for degenerative disease, trauma related to falls, chronic disease, and sports injuries. The rate of sports-related injury was significantly lower in 2020 (1.6%) than in 2019 (7.7%). In 2020, the daily hospital charge was $10,517.09 vs $11,225.32 in 2019, and the charges in the months of April and June were significantly higher in 2020 vs 2019. Both the number and rate of upper limb fractures were higher in 2020. Although the charges per day were sufficient in our institution in 2020, compared with 2019, some hospital beds were unused during this phase of the pandemic. To solve the risk of financial crisis, hospitals may increase the revenue by mixing both short-term and long-term patients’ hospital stays effectively.
